# Clinical Characteristics and Outcomes of ST-Elevation Myocardial Infarction in Young Patients: A Single-Center Experience

**DOI:** 10.7759/cureus.53688

**Published:** 2024-02-06

**Authors:** Tarique S Chachar, Husam A Noor, Nouf F AlAnsari, Abdulrahman Masood, Abdulrahman Alraee, Haitham Amin, Nooraldaem Yousif

**Affiliations:** 1 Cardiology, Mohammed Bin Khalifa Bin Salman Al Khalifa Specialist Cardiac Centre, Awali, BHR; 2 Cardiology, Bahrain Defence Force Hospital, Awali, BHR; 3 Interventional Cardiology, Mohammed Bin Khalifa Bin Salman Al Khalifa Specialist Cardiac Centre, Awali, BHR

**Keywords:** acute coronary syndrome, acute myocardial infarction, young population, coronary artery disease, primary percutaneous coronary intervention (pci), st-segment elevation myocardial infarction (stemi)

## Abstract

Objective

This study aimed to examine the clinical characteristics, risk factors, and outcomes of patients aged ≤45 years with ST-segment elevation myocardial infarction (STEMI) treated with primary percutaneous coronary intervention (pPCI).

Methods

From January 2018 to March 2020, this retrospective observational study took place at a tertiary cardiac center in Bahrain. We included patients aged ≤45 years who were admitted with STEMI and had primary percutaneous coronary intervention (pPCI).

Results

In this study, 510 patients with STEMI receiving pPCI were included, of whom 95 (18%) were younger than 45 years. The young age group had more smokers (57.9% vs. 40.5%, p = 0.003), newly diagnosed dyslipidemia (41.1% vs. 25.5%, p = 0.004), and a positive family history of early coronary artery disease (CAD) (14.7% vs. 4.3%, p = <0.001). Traditional cardiovascular risk factors, such as diabetes mellitus, systemic hypertension, and dyslipidemia, were significantly less common in young patients. Major adverse cardiovascular and cerebrovascular events (MACCE) were also significantly less common in young patients at the one-year follow-up (2.1 vs. 8.4%, p = 0.05).

Conclusion

Young patients with STEMI are more often smokers with undiagnosed dyslipidemia and have a family history of CAD. MACCE at one year is significantly lower as compared to older patients, but it is not negligible. Public health efforts are needed to reduce the prevalence of modifiable risk factors among the susceptible population.

## Introduction

Acute myocardial infarction (AMI) is a leading cause of morbidity and mortality worldwide. Primary percutaneous coronary intervention (pPCI) has revolutionized patient care, especially for those with ST-segment elevation myocardial infarction (STEMI), leading to notable increases in survival rates [1].

Despite advancements in understanding and managing coronary artery disease (CAD), the literature regarding premature CAD and AMI in younger populations remains sparse [2]. The ramifications of STEMI are particularly severe for the young, not only due to the profound psychological impact and disruption of their ability to work but also because of the extensive socioeconomic and healthcare burdens it imposes. Young patients with STEMI often bear the financial responsibility for their families, amplifying the consequences of the event on multiple dependents and national productivity [2].

The definition of 'young' in the context of AMI varies, with thresholds set at ≤40, ≤45, or ≤55 years of age [3]. While ≤45 years is generally the most accepted cutoff, the definition of premature CAD in family histories often extends to MI occurring at ≤55 years or ≤65 years for male and female relatives, respectively [4]. Despite the lack of a universal age definition, the occurrence of myocardial infarction (MI) at a young age is a significant concern, particularly in developing regions and the Middle East, where it has not been sufficiently explored [4].

The INTERHEART Study highlights the Middle East as having the youngest median age for initial MI at 51 years, markedly younger than that of North America and Western Europe, which are 59 years and 63 years, respectively [5]. Additionally, Middle Eastern and North African (MENA) countries reported the highest prevalence of individuals aged ≤40 years experiencing MI, accounting for 11%, a stark contrast to 4% in North America and 3% in Western Europe. This trend poses a critical concern for MENA countries, as escalating rates of premature MI could significantly strain national productivity and inflate healthcare costs, burdening their developing economies [5].

Although AMI is relatively rare among young individuals, its prevalence varies geographically. For instance, in young Japanese patients aged ≤40 years, the incidence is 1.6% according to the AMI-Kyoto Multicenter-Risk Study [6], compared to 5% in the Miyagi AMI Registry [7]. Other regions report varying figures, with nearly 4% in Singapore, 5.8% in the United States (for those aged between 25 and 44 years), and 1.3% in Poland (for those aged ≤40 years), as per the PL-ACS Registry [7,8].

In the landscape of cardiovascular risk factors (RF), a spectrum of modifiable elements, such as cigarette smoking, cholesterol levels, diabetes, hypertension, increased weight, dietary habits, physical inactivity, and alcohol consumption, have been identified as pivotal determinants of heart health. Specifically, cigarette smoking emerges as the predominant RF, especially among younger demographics, accentuating the need for targeted public health interventions. Notably, psychological stress and male gender are also recognized as significant RFs in this age group [9]. However, the risk profile undergoes a notable shift with age; in the elderly population, hypertension, diabetes, and dyslipidemia are recognized as the primary RFs. This variation underscores the importance of age-specific strategies in the management and prevention of cardiovascular diseases, with a particular emphasis on addressing the most critical factors: smoking, lipid abnormalities, hypertension, and diabetes, to mitigate the global burden of cardiovascular morbidity and mortality [5,9,10].

In this context, our study aimed to elucidate the prevalence of cardiovascular risk factors, as well as the clinical and angiographic characteristics and outcomes of patients aged ≤45 years who have experienced STEMI and underwent pPCI. We sought to compare these findings with those of patients aged >45 years who also underwent pPCI.

## Materials and methods

This retrospective cohort study took place at the Mohammed Bin Khalifa Specialist Cardiac Centre in the Kingdom of Bahrain from January 2018 to March 2020. In the Materials and Methods section, the inclusion criteria consisted of all patients diagnosed with acute STEMI who underwent pPCI. On the other hand, the exclusion criteria encompassed patients who received thrombolysis or medical treatment for STEMI.

Data collection and definitions

A total of 510 eligible patients admitted during the study period were recruited consecutively. A semi-structured format was used for data collection. Data on demographics, prior medical history, clinical characteristics, cardiac catheterization, angiographic characteristics, inpatient mortality, and complications were obtained from the Cardiac Centre Database. Pre-existing comorbidities and conditions that were newly diagnosed during a STEMI admission were also recorded. Outcomes after discharge and follow-up data were obtained from the hospital's electronic medical records at 30 days and 1 year. STEMI was defined in accordance with the fourth universal definition of MI [7]. Patients <45 years old were defined as the “young STEMI” group. Ethical approval was obtained from the local Ethics Committee in compliance with the Declaration of Helsinki.

Statistical analysis

Categorical variables were summarised as frequency and percentage and analyzed using the chi-square test. Continuous variables were expressed as mean and standard deviation and were analyzed using an independent sample t-test. A two-sided p-value of ≤0.05 was considered statistically significant. Data analyses were performed using EPI INFOTM version 7.2.1.0 (CDC, Atlanta, GA, USA, 2017).

## Results

In this study, 510 patients with STEMI treated with pPCI were included, of whom 95 (18%) were younger than 45 years. In the older age group, the mean standard deviation (SD) age was 57.96 ± 9.01 years, whereas in the young age group, the mean age was 38.65 ± 4.34 years.

The number of men was higher in both young (96.84%) and old age groups (86.99%); however, the difference was not statistically significant (p = 0.337).

Comparing old age vs. young age groups, a higher prevalence of conventional cardiovascular risk factors like hypertension (64.1% vs. 28.4%, p < 0.001), diabetes mellitus (49.4% vs. 31.6%, p = 0.002), dyslipidemia (57.8% vs. 33.7%, p < 0.001), and CAD (20.7 vs. 4.2%, p < 0.001) was seen. A history of active smoking and a family history of early CAD were significantly more common among young patients than old patients (57.9% vs. 40.5, p < 0.001 and 14.7% vs. 4.3, p < 0.001, respectively). Although the low-density lipoprotein level (LDL) cholesterol level was lower compared with the old age group, newly diagnosed dyslipidemia was more common in the young group (41.1% vs. 25.5%, p = 0.004). Furthermore, the mean creatine phosphokinase level was significantly higher in the young group than in the old group (3034 vs. 2345, p = 0.02). No significant difference was seen in other biochemical parameters and procedural characteristics among the study groups, except for right ventricular infarction, which was less likely to be involved in the setting of inferior wall STEMI in the young age group than in the old age group (0% vs. 6.7%, p = 0.018) (Tables 1, 2).

**Table 1:** Baseline characteristics and biochemical parameters for the study population

Variables	Young age group (N=95)	Old age group (N=415)	P value
Male	92 (96.8%)	361 (86.9%)	0.10
Hypertension	27 (28.4%)	266 (64.1%)	<0.001
Diabetes mellitus	30 (31.6%)	205 (49.4%)	0.002
Dyslipidemia	32 (33.7%)	240 (57.8%)	<0.001
New dyslipidemia	39 (41.1%)	106 (25.5%)	0.004
Smoking	55 (57.9%)	168 (40.5%)	0.003
Family history	14 (14.7%)	18 (4.3%)	<0.001
Coronary artery disease	4 (4.2%)	86 (20.7%)	<0.001
Chronic kidney disease	3 (3.2%)	28 (6.7%)	0.279
Peripheral vascular disease	0 (0%)	8 (1.9%)	0.365
Chronic obstructive airway disease	1 (1.1%)	3 (0.7%)	0.752
Stroke	1 (1.1%)	13 (3.1%)	0.441
Hemoglobin g/dl	148.65 ± 17.72	140.07 ± 20.97	<0.001
Creatinine µmol/l	81.29 ± 42.68	95.35 ± 105.28	0.202
Hemoglobin A1C %	7.37 ± 2.66	9.36 ± 36.07	0.598
Total cholesterol (TC) mmol/L	5.21 ± 1.55	5.91 ± 26.62	0.796
Low-density lipoprotein (LDL) mmol/L	3.92 ± 1.52	4.96 ± 19.79	0.612
High-density lipoprotein (HDL) mmol/L	1.94 ± 9.55	1.41 ± 7.26	0.544
Triglyceride (TG) mmol/L	2.02 ± 1.36	2.31 ± 10.22	0.782
Troponin-I ng/ml	15.82 ± 8.87	17.21 ± 67.25	0.845
Creatine phosphokinase (CPK) U/L	3034.72 ± 2612.07	2345.3 ± 2712.31	0.026

**Table 2:** Procedural characteristics of the study population STEMI: ST-elevation myocardial infarction, POBA: percutaneous old balloon angioplasty, TIMI: thrombolysis in myocardial infarction

Parameters	Young age group	Old age group	P value
Cardiogenic shock	7 (7.4%)	34 (8.2%)	0.954
Inotropic support	9 (9.5%)	49 (11.8%)	0.640
Intra-aortic balloon pump	6 (6.3%)	29 (7%)	0.927
Cardiopulmonary resuscitation	8 (8.4%)	27 (6.5%)	0.659
Mechanical ventilation	5 (5.3%)	23 (5.5%)	0.887
Arrhythmia	13 (13.7%)	71 (17.1%)	0.510
Anterior STEMI	51 (53.7%)	192 (46.3%)	0.233
Inferior STEMI	40 (42.1%)	195 (47%)	0.455
Lateral STEMI	9 (9.5%)	26 (6.3%)	0.373
Right ventricular infarction	0 (0%)	28 (6.7%)	0.018
Ejection fraction	40.43 ±10.10	41.98 ± 10.69	0.198
Radial access	86 (90.5%)	352 (84.8%)	0.201
Thrombus aspiration,	38 (40%)	125 (30.1%)	0.082
Glycoprotein IIb IIIa	21 (22.1%)	67 (16.1%)	0.216
Drug-eluting stent	86 (90.5%)	376 (90.6%)	0.278
Bare metal stent	6 (6.3%)	16 (3.9%)	0.278
Drug-eluting balloon	0 (0%)	10 (2.4%)	0.278
POBA	3 (3.2%)	25 (6%)	0.278
TIMI III flow	94 (98.9%)	407 (98.1%)	0.879
Left main disease	6 (6.3%)	21 (5.1%)	0.811
Single vessel disease	70 (73.7%)	283 (68.2%)	0.565
Double vessel disease	18 (18.9%)	98 (23.6%)	0.565
Triple vessel disease	7 (7.4%)	34 (8.2%)	0.565
Aspirin	93 (97.9%)	401 (96.6%)	0.198
Brilinta	28 (29.5%)	151 (36.4%)	0.314
Plavix	64 (67.4%)	254 (61.2%)	0.240
Oral anticoagulant	12 (12.6%)	34 (8.2%)	0.221

At the 30-day follow-up, there was no statistically significant difference in the primary outcomes of cardiovascular mortality, nonfatal myocardial infarction (MI), stroke, re-admission, and major adverse cardiovascular and cerebrovascular events (MACCE). At the one-year follow-up, MACCE was statistically higher in the old age group (2.1% vs. 8.4%, p = 0.05) (Table 3).

**Table 3:** Clinical outcomes at the 30-day and 1-year follow-ups

Follow-up	Parameter	Young age group	Old age group	P value
30 days	Cardiovascular mortality	1 (1.1%)	8 (1.9%)	0.882
	Re-admission	4 (4.2%)	20 (4.8%)	0.971
	Non-fatal myocardial infarction	1 (1.1%)	5 (1.2%)	0.687
	Stroke	1 (1.1%)	1 (0.2%)	0.817
	Major adverse cardiovascular and cerebrovascular events (MACC)	1 (1.1%)	0 (0%)	0.420
1 year	Cardiovascular mortality	2 (2.1%)	13 (3.1%)	0.843
	Re-admission	4 (4.2%)	42 (10.1%)	0.106
	Non-fatal myocardial infarction	1 (1.1%)	9 (2.2%)	0.766
	Stroke	1 (1.1%)	1 (0.2%)	0.817
	Major adverse cardiovascular and cerebrovascular events (MACCE)	2 (2.1%)	35 (8.4%)	0.05

## Discussion

Young people's stressful lifestyles, fast pace, overwork, smoking, and overeating induce internal environment changes, such as coronary atherosclerosis, which enhance AMI incidence [11]. The pathophysiology of MI in individuals younger than 45 years is multifaceted and can be broadly classified into four primary categories, elucidating the diverse etiological landscape of this condition in a younger cohort. First, atherosclerotic CAD stands out as a traditional contributor, underscoring the pivotal role of lipid accumulation and plaque formation in precipitating cardiac events. Second, non-atherosclerotic abnormalities in the coronary arteries, encompassing a range of anatomical and functional aberrations, highlight the complexity beyond the conventional atherosclerotic pathways. Third, recreational substance use, particularly drugs that exert potent vasoactive effects or induce cardiac stress, emerges as a critical factor, especially given its prevalence and impact among the younger population. Finally, conditions that make blood more likely to clot, whether they are inherited or acquired, add to this range by making people more likely to have thrombotic events. This makes the pathophysiology of MI in this age group even more complicated [12].

Modifiable cardiovascular risk factors have garnered significant attention in research on premature AMI, primarily because of their pivotal role in secondary prevention strategies following an acute coronary event [13,14]. According to prior research, it is noteworthy that more than 90% of patients with premature AMI have at least one modifiable risk factor, primarily smoking, obesity, dyslipidemia, and hypertension. This high prevalence underscores the criticality of targeted interventions and lifestyle modifications in the patient cohort [13,14]. The results of our study add to this story by showing that smoking history, a family history of premature CAD, and newly diagnosed dyslipidemia are the main risk factors for STEMI in young people. This shows how important it is to keep a closer eye on these factors and take action to change them in order to lower the number of cases and occurrences of premature AMI.

Matsis K. et al. [15] found that young individuals had a lower prevalence of chronic disorders, such as hypertension, diabetes mellitus, and stroke, than older patients [16]. Chronic disease prevalence was likewise consistent with these trends in our study.

Obeidat et al.'s pioneering work in the first Jordanian Percutaneous Coronary Intervention Registry revealed a striking prevalence of the male gender among STEMI patients aged below 45 years, with a substantial 96% representation compared to 82% in those above 45 years. This demographic trend aligns remarkably with the findings of our study, where an overwhelming 96.8% of young male patients comprised the STEMI demographic in the under-45 age group [17].

Cigarette smoking emerges as the predominant and most prevalent risk factor (RF) among younger individuals in contrast with the elderly population, where hypertension, diabetes, and dyslipidemia take precedence as the most common RFs [5,15]. Notably, smoking stands out as the solitary element among these risk factors that can be entirely eradicated. The act of smoking tobacco accelerates the progression of atherosclerosis through mechanisms such as the diminution of tissue oxygenation, damage to the vascular endothelium, and the amplification of sympathetic nervous system activity. Also, smoking makes things worse by increasing the activity of platelets that clump together. This makes it easier for clots to form inside blood vessels, which is a key part of how acute coronary syndromes and other vascular events happen. This dual impact of smoking - both as a direct contributor to atherosclerosis and as an enhancer of thrombotic processes - underscores its pivotal role in cardiovascular risk, especially among the younger population, and highlights the profound benefits that can be achieved through smoking cessation [18].

In the context of our region, the prevalence of smoking among the young population has been a focal point of recent research endeavors. A notable study by Haitham Sakr et al. highlighted that 52% of the young population in Saudi Arabia are smokers, a statistic that offers critical insight into the behavioral risk patterns within this demographic [19]. Our study sheds further light on this issue, reporting a smoking prevalence of 57.9% among the young cohort, a figure that, while lower than some of the international data reported, notably exceeds the contemporary figures from within Saudi Arabia. An in-depth analysis of the local population's characteristics reveals a clear difference in smoking patterns across genders. According to a regional epidemiological survey, men had a far higher tendency to smoke (33.4%) compared to women (7.1%) [20]. This gender-specific prevalence not only reflects the societal and cultural dynamics influencing health behaviors but also underscores the need for targeted public health strategies to address and mitigate the impact of smoking, particularly among the male populace. Among the younger participants in our study, a significant majority (71%) exhibited dyslipidemia. Fifty-four point nine percent (54.9%) of patients were unaware of their elevated cholesterol levels or any prior treatment they had received for it. Hence, it is imperative to do early screening for dyslipidemia in high-risk individuals such as young smokers or those with a positive family history of premature CAD.

The infarct size was bigger based on creatine phosphokinase (3034 vs. 2345, p = 0.02), but young people appear to experience few MACCE. To draw conclusions or inferences regarding this observation, it is essential to conduct a multivariate analysis. This involves identifying important factors and determining their significance.

Limitations

Our study was conducted at a single center, which may limit the generalizability of the findings to a broader population. It used a retrospective observational design, which is prone to various biases and may not establish causation between the variables being studied. The relatively small sample size used in the study may affect the statistical power and precision of the results, making it difficult to draw firm conclusions. There could be selection bias in the study, meaning that the participants included may not be representative of the entire population being studied. The article acknowledges that data on newly diagnosed diabetes mellitus and hypertension were unavailable and were not included, potentially limiting the comprehensiveness of the findings. The sample sizes of the groups compared in the study were disparate, which may introduce imbalance or unequal statistical power between the groups.

## Conclusions

In conclusion, this study highlights the importance of addressing modifiable risk factors in young patients with STEMI. The findings indicate that a significant proportion of these patients are smokers with undiagnosed dyslipidemia and a family history of CAD. This underscores the need for public health efforts to reduce the prevalence of these risk factors among this vulnerable population. Moreover, the study reveals that while the major adverse cardiac events at one year and MACCE were lower in the younger age group as compared to older patients, they were still noticeable. This suggests that even though STEMI is relatively rare in the younger population, the impact on long-term cardiovascular health should not be ignored. Efforts should be focused on early detection and intervention, as well as educating young individuals about the importance of adopting a healthy lifestyle to prevent future cardiac events.

Overall, the study emphasizes the urgency of targeted interventions and public health campaigns to reduce the burden of STEMI such as smoking, dyslipidemia, and family history of CAD. By doing so, we can potentially improve the outcomes and quality of life for this susceptible population. Timely interventions and widespread awareness are crucial in safeguarding the cardiovascular health of young individuals and reducing the overall burden of cardiovascular disease.

## References

[1] Yousif N, Chachar TS, Subbramaniyam S, Vadgaonkar V, Noor HA (2021). Safety and feasibility of 48 h discharge after successful primary percutaneous coronary intervention. J Saudi Heart Assoc.

[2] Shah N, Kelly AM, Cox N, Wong C, Soon K (2016). Myocardial infarction in the “young”: risk factors, presentation, management and prognosis. Heart Lung Circ.

[3] Samir A, Almahjori M, Zarif B (2023). Characterization of features and outcomes of young patients (< 45 years) presenting with ST-segment elevation myocardial infarction. Egypt Heart J.

[4] Visseren FL, Mach F, Smulders YM (2021). 2021 ESC Guidelines on cardiovascular disease prevention in clinical practice. Eur Heart J.

[5] Yusuf S, Hawken S, Ounpuu S (2004). Effect of potentially modifiable risk factors associated with myocardial infarction in 52 countries (the INTERHEART study): case-control study. Lancet.

[6] Shiraishi J, Kohno Y, Yamaguchi S (2005). Acute myocardial infarction in young Japanese adults. Clinical manifestations and in-hospital outcome. Circ J.

[7] Cui Y, Hao K, Takahashi J (2017). Age-specific trends in the incidence and in-hospital mortality of acute myocardial infarction over 30 years in Japan — report from the Miyagi AMI Registry Study —. Circ J.

[8] Muhammad AS, Ashraf T, Mir A (2020). Comparative assessment of clinical profile and outcomes after primary percutaneous coronary intervention in young patients with single vs multivessel disease. World J Cardiol.

[9] Vaccarino V, Sullivan S, Hammadah M (2018). Mental stress-induced-myocardial ischemia in young patients with recent myocardial infarction: sex differences and mechanisms. Circulation.

[10] Tung BW, Ng ZY, Kristanto W (2021). Characteristics and outcomes of young patients with ST segment elevation myocardial infarction undergoing primary percutaneous coronary intervention: retrospective analysis in a multiethnic Asian population. Open Heart.

[11] Yunyun W, Tong L, Yingwu L (2014). Analysis of risk factors of ST-segment elevation myocardial infarction in young patients. BMC Cardiovasc Disord.

[12] Sood A, Singh A, Gadkari C (2023). Myocardial infarction in young individuals: a review article. Cureus.

[13] Yandrapalli S, Nabors C, Goyal A, Aronow WS, Frishman WH (2019). Modifiable risk factors in young adults with first myocardial infarction. J Am Coll Cardiol.

[14] Leifheit-Limson EC, D'Onofrio G, Daneshvar M (2015). Sex differences in cardiac risk factors, perceived risk, and health care provider discussion of risk and risk modification among young patients with acute myocardial infarction: the VIRGO Study. J Am Coll Cardiol.

[15] Matsis K, Holley A, Al-Sinan A, Matsis P, Larsen PD, Harding SA (2017). Differing clinical characteristics between young and older patients presenting with myocardial infarction. Heart Lung Circ.

[16] Thygesen K, Alpert JS, Jaffe AS, Chaitman BR, Bax JJ, Morrow DA, White HD (2018). Fourth universal definition of myocardial infarction (2018). Glob Heart.

[17] Obeidat OS, Makhamreh H, Hammoudeh A (2021). Clinical characteristics and prognosis of young Middle Eastern adults with ST-elevation myocardial infarction: one-year follow-up. Heart Views.

[18] Rywik S, Kupść W, Piotrowski W (2005). Multicenter national Polish population health status tests--WOBASZ project. Establishment of methods and logistics [Article in Polish]. Kardiol Pol.

[19] Sakr H, Azazy AS, Hillani A (2021). Clinical profiles and outcomes of acute ST-segment elevation myocardial infarction in young adults in a tertiary care center in Saudi Arabia. Saudi Med J.

[20] (2010). Ministry of Health Bahrain. National non-communicable diseases risk factors survey Kingdom of Bahrain. https://www.moh.gov.bh/Content/Files/Publications/x_2812013135226.pdf.

